# Nipah virus: epidemiology, pathology, immunobiology and advances in diagnosis, vaccine designing and control strategies – a comprehensive review

**DOI:** 10.1080/01652176.2019.1580827

**Published:** 2019-04-22

**Authors:** Raj Kumar Singh, Kuldeep Dhama, Sandip Chakraborty, Ruchi Tiwari, Senthilkumar Natesan, Rekha Khandia, Ashok Munjal, Kranti Suresh Vora, Shyma K. Latheef, Kumaragurubaran Karthik, Yashpal Singh Malik, Rajendra Singh, Wanpen Chaicumpa, Devendra T. Mourya

**Affiliations:** a ICAR-Indian Veterinary Research Institute, Bareilly, India;; b Division of Pathology, ICAR-Indian Veterinary Research Institute, Bareilly, India;; c Department of Veterinary Microbiology, College of Veterinary Sciences & Animal Husbandry, West Tripura, India;; d Department of Veterinary Microbiology and Immunology, College of Veterinary Sciences, Deen Dayal Upadhayay Pashu Chikitsa Vigyan Vishwavidyalay Evum Go-Anusandhan Sansthan (DUVASU), Mathura, India;; e Biomac Life Sciences Pvt Ltd., Indian Institute of Public Health Gandhinagar, Gujarat, India;; f Department of Biochemistry and Genetics, Barkatullah University, Bhopal, India;; g Wheels India Niswarth (WIN) Foundation, Maternal and Child Health (MCH), University of Canberra, Gujarat, India;; h Central University Laboratory, Tamil Nadu Veterinary and Animal Sciences University, Chennai, India;; i Division of Biological Standardization, ICAR-Indian Veterinary Research Institute, Bareilly, India;; j Center of Research Excellence on Therapeutic Proteins and Antibody Engineering, Department of Parasitology, Faculty of Medicine, Siriraj Hospital, Mahidol University, Bangkok, Thailand;; k National Institute of Virology, Ministry of Health and Family Welfare, Govt of India, Pune, India

**Keywords:** Nipah virus (NiV), bats, diagnosis, encephalitis, epidemiology, pathology, prevention, control, vaccines, therapeutics, zoonosis

## Abstract

Nipah (Nee-pa) viral disease is a zoonotic infection caused by Nipah virus (NiV), a paramyxovirus belonging to the genus *Henipavirus* of the family Paramyxoviridae. It is a biosafety level-4 pathogen, which is transmitted by specific types of fruit bats, mainly *Pteropus* spp. which are natural reservoir host. The disease was reported for the first time from the Kampung Sungai Nipah village of Malaysia in 1998. Human-to-human transmission also occurs. Outbreaks have been reported also from other countries in South and Southeast Asia. Phylogenetic analysis affirmed the circulation of two major clades of NiV as based on currently available complete N and G gene sequences. NiV isolates from Malaysia and Cambodia clustered together in NiV-MY clade, whereas isolates from Bangladesh and India clusterered within NiV-BD clade. NiV isolates from Thailand harboured mixed population of sequences. In humans, the virus is responsible for causing rapidly progressing severe illness which might be characterized by severe respiratory illness and/or deadly encephalitis. In pigs below six months of age, respiratory illness along with nervous symptoms may develop. Different types of enzyme-linked immunosorbent assays along with molecular methods based on polymerase chain reaction have been developed for diagnostic purposes. Due to the expensive nature of the antibody drugs, identification of broad-spectrum antivirals is essential along with focusing on small interfering RNAs (siRNAs). High pathogenicity of NiV in humans, and lack of vaccines or therapeutics to counter this disease have attracted attention of researchers worldwide for developing effective NiV vaccine and treatment regimens.

## 1. Introduction

Viral diseases like Avian/bird flu, Swine flu, Middle East respiratory syndrome coronavirus (MERS-CoV), Severe acute respiratory syndrome (SARS), Crimean-Congo haemorrhagic fever (CCHF), Lassa fever, Rift Valley fever (RVF), Marburg virus disease, Ebola, Zika, Nipah and Henipaviral diseases pose considerable risk of an international public health emergency, when these spread rapidly (Rizzardini et al. 2018). After the recent emergency situations created by Ebola and Zika virus during past five years (Singh et al. 2016, 2017), now Nipah virus disease outbreaks have created panic in the public. Ebola virus disease (EVD) outbreaks and epidemics (2014–2016) led a massive mobilization of researchers to seek new technologies in terms of developing efficient and rapid diagnostics, vaccines, therapies and drug targets to combat EVD and save lives of large human population across the globe. Like Zika, scientists are on the way to counter Nipah virus.

Nipah (Nee-pa) viral disease is a zoonotic infection and an emerging disease caused by Nipah virus (NiV), an RNA virus of the genus *Henipavirus,* family Paramyxoviridae, which is transmitted by specific types of fruit bats, mainly *Pteropus* spp. (Halpin et al. 2000; Vandali and Biradar, 2018). NiV is a highly fatal virus posing potential threat to global health security. The *Pteropus* bats, *viz*., *P. vampyrus, P. hypomelanus, P. lylei* and *P. giganteu,* were associated with outbreaks of the Nipah viral disease in various countries of South and Southeast Asia, including Bangladesh, Cambodia, East Timor, Indonesia, India, Malaysia, Papua New Guinea, Vietnam and Thailand (Hayman et al. 2008; Sendow et al. 2010; Wacharapluesadee et al. 2010; Halpin et al. 2011; Hasebe et al. 2012; Yadav et al. 2012; Field et al. 2013; de Wit and Munster, 2015a; Majid and Majid Warsi 2018). Fruit bats are the major reservoirs of the virus and it is the contact with such bats (infected) or intermediate hosts like pigs which are responsible for infection in man. It is to be remembered that various biologic as well as genetic features of various paramyxoviruses are retained by Nipah virus (Bellini et al. 2005). Dependence on animal rearing as a source of additional income in many Asian countries is a predisposing factor for emergence of novel zoonoses like Nipah (Bhatia and Narain 2010). Various studies reported that major factor responsible for emergence of NiV was thorough interaction between wildlife reservoir particularly fruit bats of the *Pteropus* spp. with animal population reared and managed under intensive conditions (Daszak et al. 2013). The high fatality rate associated with Nipah disease and the lack of efficacious treatment and vaccines against it, classify it as a global threat (Epstein et al. 2006; Rahman and Chakraborty 2012). The disease was recognized for the first time in 1998 in Kampung Sungai Nipah village, state of Perak, Malaysia. The causative agent was characterized and since then has been named as “Nipah virus (NiV)”. The zoonotic potential of NiV was unknown before 1999 till Malaysia experienced Nipah viral outbreak. Such an outbreak had created alarming situation in the public health community globally as far as the potential of severe pathogenicity as well as viral distribution in widespread fashion are concerned (Chua 2012). Considerable uncertainty exists about the patterns of Nipah virus circulation in bats and the epidemiological factors associated with its spill-over into pigs and horses (McCormack 2005).

Encephalitis (acute) along with high mortality is the main manifestation of infection due to NiV. Apart from this there may be development of pulmonary illness and sometimes the infection may be asymptomatic in nature (Kitsutani and Ohta 2005). Myoclonus (segmental) along with tachycardia may become evident. The involvement of brain stem, which locates the major vital centres, is probably responsible for death and mortality may vary between 32% and 92%. From a diagnostic point of view serology is quite helpful but discrete, high signal lesions can be visualized best by fluid-attenuated inversion recovery (FLAIR) where the effect of cerebrospinal fluid (CSF) is reduced, so that an enhanced MRI image can be obtained (Arif et al. 2012). Nipah virus was first isolated in 1999 (Farrar 1999; Rahman et al. 2012). Gene sequencing of the isolates showed that the outbreak involved two different NiV strains, probably with different origins (AbuBakar et al. 2004). The clinical signs and symptoms of the NiV disease include fever along with laboured breathing, cough and headache. Encephalitis along with seizures are the complications involved (Broder et al. 2013). Survivors of NiV infection develop symptoms of neurological malfunction such as encephalopathy, cerebral atrophy, change in behavior, ocular motor palsies, cervical dystonia, weakness and facial paralysis, which remain for several years (Sejvar et al. 2007). Despite an increasing risk, rigorous studies that collate data from Nipah infections of pigs, bats and humans have been scarce (Hsu et al. 2004; Chadha et al. 2006; Pulliam et al. 2012). Serosurveillance studies in multiple host species may yield important insights into NiV epidemiology (Weingartl et al. 2009; Li et al. 2010; Rockx et al. 2010; Pallister et al. 2011; Fischer et al. 2018).

The NiV belongs to the *Henipavirus* genus under the family Paramyxoviridae. This genus alsocontains Cedar virus (CedPV) and Hendra virus (HeV). Molecular studies have significantly improved our understanding of the genetic diversity of Henipaviruses (Wang et al. 2001; Rockx et al. 2012).The almost annual occurrence of Henipaviruses in South-Eastern Asia and Australia since the mid 1990s is noteworthy. In Australia alone 48 cases of Hendra viruses and in south eastern parts of Asia 12 outbreaks of Nipah viruses have been reported which not only hit the health sector but also the economic stability of these nations (Aljofan, 2013). There have been a total 639 human cases of NiV infection reported from Bangladesh (261 cases), India (85 cases), Singapore (11 cases), Philippines (17 cases) and Malaysia (265 cases), with a mortality rate of about 59% (Ang et al. 2018). This points to the survival efficiency of NiV in nature and the history on its species jumping/host adaptation pattern adds to the public health concerns posed by this virus. Detailed studies and clinical therapeutic trials on various animal models such as guinea pigs, hamsters, ferrets, cats, pigs and African green monkeys are being investigated for Henipaviruses (Geisbert et al. 2012). The Nipah disease outbreak in 2001 in Siliguri and latest in Kerala have emphazised the need for an efficacious vaccine against it. Moreover the virus imposes threat to health of public (Sharma et al. 2018). Enhanced monitoring and surveillance for Nipah infection and the development of an efficacious vaccine are the needs of the hour.

This review discusses in detail the NiV biology, its transmission and epidemiology, pathology, and advances in diagnosis, vaccine designing, and suitable prevention and control studies to be adopted to counter this emerging pathogen.

## 2. Nipah virus

Nipah virus (NiV) is a paramyxovirus (*Henipavirus* genus, *Paramyxovirinae* subfamily, *Paramyxoviridae* family, order *Mononegavirales*), an emerging virus that can cause severe respiratory illness and deadly encephalitis in humans. It is a negative sense, single-stranded, nonsegmented, enveloped RNA virus possessing helical symmetry. The RNA genome, from the 3´-5´, contains consecutive arrangement of six genes, *viz*., nucleocapsid (N), phosphoprotein (P), matrix (M), fusion glycoprotein (F), attachment glycoprotein (G) and long polymerase (L). The N, P and L attached to the viral RNA forming the virus ribonucleoprotein (vRNP). F and G proteins are responsible for cellular attachment of the virion and subsequent host cell entry (Ternhag and Penttinen 2005; Ciancanelli and Basler 2006; Bossart et al. 2007). The newly produced precursor F protein (F0) is cleaved into two subunits, *viz*., F1 and F2, by host protease. The fusion peptide of the virus contained in the F1 subunit drives the viral and host cellular membrane fusion for the virus entry (Eaton et al. 2006). The virus M protein mediates morphogenesis and budding. Antibody to the G protein is essential for neutralization of the NiV infectivity (Bossart et al. 2005; White et al. 2005). It is quite noteworthy that through the coordinated efforts of the fusion (F) (class I) and attachment (G) glycoproteins the target cell (i.e. host cell) is entered upon after binding by the enveloped Henipaviruses including NiV. Interactions between Class B ephrins (viral receptors) on host cells and the NiV glycoprotein (G) trigger conformational changes in the latter, leading to activation of F glycoprotein and membrane fusion (Steffen et al. 2012). It is believed that the strategies of replication as well as fusion of the ephrin receptors are responsible for greater pathogenicity of these viruses. Multiple accessory proteins encoded by Henipaviruses aid in host immune evasion(Marsh and Wang 2012).

**Figure 1.**
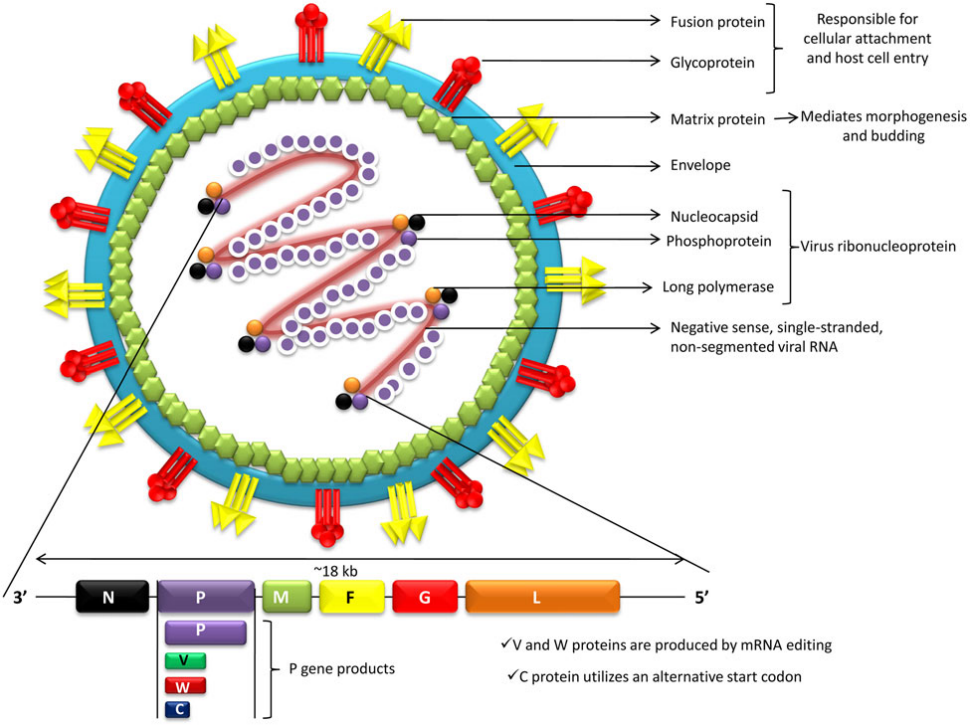
Structure of Nipah virus.

NiV infects its host cells *via* two glycoproteins, i.e. G and F proteins. The G glycoprotein mediates attachment to host cell surface receptors and the fusion (F) protein makes fusion of virus-cell membranes for cellular entry. The G protein of NiV binds to host ephrin B2/3 receptors and induces conformational changes in G protein that trigger the F protein refolding (Liu et al. 2015). Wong et al. (2017) have demonstrated that monomeric ephrinB2 binding leads to allosteric changes in NiV G protein that pave the way to its full activation and receptor-activated virus entry into the host cells. Recently, viral regulation of host cell machinery has been revealed to target nucleolar DNA-damage response (DDR) pathway by causing inhibition of nucleolar Treacle protein that increases *Henipavirus* (Hendra and Nipha virus) production (Rawlinson et al. 2018). A diagrammatic structure of Nipah virus is depicted in Figure 1.

Nipah virus can survive for up to 3 days in some fruit juices or mango fruit, and for at least 7 days in artificial date palm sap (13% sucrose and 0.21% BSA in water, pH 7.0) kept at 22 °C. The virus has a half-life of 18 h in the urine of fruit bats. NiV is relatively stable in the environment, and remains viable at 70 °C for 1 h (only the viral concentration will be reduced). It can be completely inactivated by heating at 100 °C for more than 15 min (de Wit et al. 2014). However, the viability of the virus in its natural environment may vary depending on the different conditions. NiV can be readily inactivated by soaps, detergents and commercially available disinfectants such as sodium hypochlorite (Hassan et al. 2018).

NiV infection produces severe respiratory symptoms in pigs compared to humans. A rapid spread of NiV is seen in human airway epithelia which express high levels of the NiV entry receptor ephrin-B2, and the expression levels vary between cells of different donors (Sauerhering et al. 2016). NiV infection upregulates IFN-λ in human respiratory epithelial cells. IFN-λ pretreatment can proficiently demonstrate antiviral activity by hindering NiV replication and thus variations in its receptor expression can participate in a useful role in NiV replication kinetics in different donors (Sauerhering et al. 2017). The NiV V protein, which is one of the three accessory proteins encoded by the viral P gene, plays crucial role in pathogenesis of the virus in experimental infection in hamster. NiV V protein has been shown to increase the level of a host protein UBXN1 (UBX domain-containing protein 1, a negative controller of RIG-I-like receptor signaling) by restraining its proteolysis and thus regulating (suppressing) induction of innate interferons (Uchida et al. 2018). Analyzing viral proteins, their structure and biological functions would help in designing possible strategies for designing appropriate drugs and vaccines (Sun et al. 2018). A variety of cellular machinery is recruited by matrix protein of NiV in order to scaffold the viral structure as well as facilitate the assembly and co-ordinatevirion budding. The matrix protein also highjacks ubiquitination pathways to facilitate transient nuclear localization. It is crucial to note that amongst the matrix proteins there is conservation of the molecular details of the virus (Watkinson and Lee 2016). Production of viral RNA as well as regulation of viral polymerase activity is governed by overexpression of the nucleocapsid protein of NiV. There is inhibition of transcription (of viral specific proteins) due to overexpression of such protein but definitely synthesis of genome of the virus is increased. Ultimately, the progeny of the virus is inhibited due to the bias of the activity of polymerase towards production of genome (Ranadheera et al. 2018). Super-resolution microscopy revealedrandom distribution of F as well as G proteins on the NiV plasma membrane irrespective of the presence of matrix (M) protein. Virus like particles (VLPs) are formed due to the assembly of M molecules at the plasma membrane (Liu et al. 2018). G protein recruitment into VLPs is augmented by formation of viral particles that are driven by F, M as well as M/F. Such studies on viral proteins aid in improving the knowledge regarding the process of virus assembly which can ultimately spearhead researchers to design effective and specific therapeutics (Johnston et al. 2017). Further for developing prophylactic as well as therapeutic agents it is necessary to know the interaction between host and NiV. The microRNA processing machinery along with the PRP19 complex are the host targets of the virus. The p53 control along with expression of genesisgets altered by W protein of the virus. Affinity purification coupled with mass spectrometry has helped to identify interaction between the human as well as NiV proteins (Martinez-Gil et al. 2017). VLPs consisting of M, G and F proteins have been produced in human-derived cells, and have been characterized by liquid chromatography and mass spectrometry (Vera-Velasco et al. 2018).

## 3. Transmission of the Nipah virus

Bats serve as reservoir hosts for several high risk pathogens, including Nipah, rabies and Marbug viruses. Such viruses are not associated with any significant pathological changes in the bat population (O’Shea et al. 2014; Schountz 2014). Detailed studies are needed to understand the mechanisms of NiV transmission from bats-to-pigs, pigs-to-man, and from date palm sap to human and viral circulation between fruit bats, pigs and human beings. Fruit bats act as natural reservoir of Nipah viruses and among various outbreaks documented from different geographical parts of the globe these bats have been associated in one or other way for transmission of the virus and associated infection (Clayton et al. 2016; Yadav et al. 2018). From bats, the virus has crossed its species-barrier frequently to several other species including man through spilled over transmission, but with limited transmission from person to person thereafter (Gurley et al. 2017). Transmission of NiV to man occurs mainly in places where man, pigs and bats come in close proximity. People rear pigs for economic benefits and fruit bearing trees are also cultivated in and around the farm for shade. Bats of *Pteropus* spp. which are NiV reservoirs, are attracted by the fruits, hence NiV gets spilled over to pigs/animals and also to man. Infected pig meat travels across continents which led to transmission of virus from animals in one part of the world to people in another part of globe. This combination of close surroundings of fruiting trees, fruits- like date palm, fruit bats, pigs and man altogether form the basis of emergence and spread of new deadly zoonotic virus infections like Nipah (Pulliam et al. 2012).

**Figure 2.**
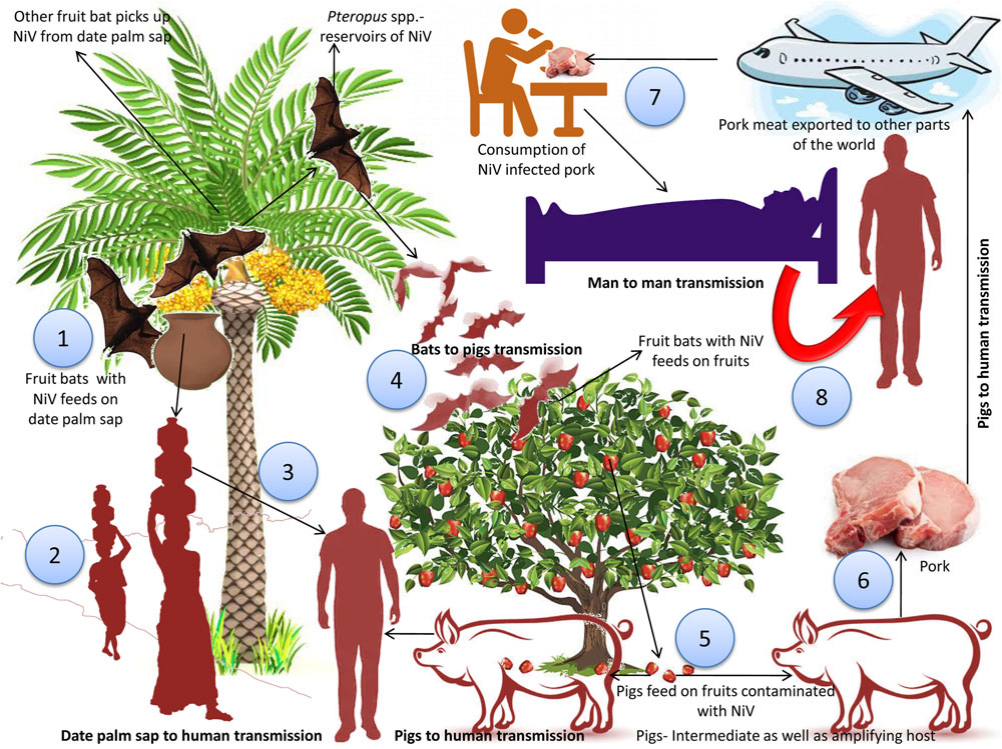
Transmission of the Nipah virus. 1. Fruit bats acts as natural reservoir of Nipah viruses. Fruit bats with NiV feeds on date palm sap. Virus can survive in solutions that are rich in sugar, *viz*., fruit pulp. 2. Virus transmitted to human through the consumption of date palm sap. 3. Fruit bats of *Pteropus* spp. which are NiV reservoirs visited such fruit trees and got opportunity to naturally spill the drop containing virus in the farm to contaminate the farm soil and fruits. 4. Contaminated fruits are consumed by pigs and other animals. Pigs act as intermediate as well as amplifying host. Combination of close surroundings of fruiting trees, fruits-like date palm, fruit bats, pigs and human altogether form the basis of emergence and spread of new deadly zoonotic virus infection like Nipah. 5. Pork meat infected with NiV are exported to other parts. 6. Consumption of infected pork can act as a source of infection to human. 7. Close contact with NiV affected human can lead to spread of NiV to other persons.

NiV transmission occurs via consumption of virus-contaminated foods and contact with infected animals or human body fluids. Risk factors include close proximity viz., touching, feeding or attending virus infected person, thus facilitating contact to droplet NiV infection. Recently, experimental studies with aerosolized NiV in Syrian hamsters revealed that NiV droplets (aerosol exposure) might play a role in transmitting NiV during close contact (Escaffre et al. 2018). Three transmission pathways of the Nipah virus have been identified after investigation carried out in Bangladesh. Consumption of freshdate palm sap is the most frequent route, with the consumption of tari (fermented date palm juice) being a potential pathway of viral transmission. NiV infection associated with tari can be prevented by prevention of the access of bat to date palm sap (Islam et al. 2016). Studies using infrared camera revealed that the date palm trees are often visited bats like *Pteropus giganteus* and during the process of collection of the sap, bats lick them. The virus can survive for days in sugar-rich solutions, viz., fruit pulp (Fogarty et al. 2008; Khan et al. 2008). The Nipah viral outbreak reported from Tangail district, Bangladesh was found to be associated with drinking of raw date palm sap. Notably, symptoms have been recognized in patients in Bangladesh during the season of collection of date palm sap, i.e. during December to March (Luby et al. 2006, 2009). Data also revealed high seroprevalence of anti-Nipah viral antibodies among *Pteropus*spp. This is suggestive of the fact that the virus has undergone adaptation well enough to get transmitted among *Pteropus* bats. The modes of transmission of the Nipah virus are depicted in Figure 2.

Research based on detailed interview of NiV infection survivors, medical practitioners and caretakers conducted in Bangladesh during May–December, 2004 showed that transmission occurred through both ways from bats-to-man and man-to-man. Some communities were of opinion that NiV infections were due to supernatural or mystic powers and hence spiritual strategies were looked for the correction of illness. Result of investigation suggested need of collaborative prevention and control measures by spreading the awareness campaigns (Blum et al. 2009). Secondary transmission between humans was observed during the outbreaks (Homaira et al. 2010a; Sazzad et al. 2013; Hegde et al. 2016). NiV shed from *Pteropus* spp. may infect either single or multiple individuals and the chain of transmission may then continue in an epidemic form by person-to-person transmission (Gurley et al. 2007). Risk factors associated with the infection include patient handling and contact with the secretion of infected person (Stone 2011).

Investigations during NiV outbreaks in Malaysia revealed that pigs are the intermediate as well as amplifying hosts for the virus (Nor et al. 2000; de Wit and Munster 2015b). In Bangladesh, domestic animals represented another route of transmission of NiV. Foraging for fruits (contaminated with infectious saliva) was observed among domestic animals in Bangladesh. There has been a report of spread of the disease from sick cows during the year 2001 in a place called Meherpur in Bangladesh (Hsu et al. 2004). Illness acquired from pigs or saliva of goats and secretions of bats infected with Nipah virus has also been recorded in Naogaon (International Centre for Diarrhoeal Disease Research, Bangladesh; ICDDRB 2003; Montgomery et al. 2008; Hughes et al. 2009). In ferrets, systemic disease was induced when the animals are exposed to certain doses of NiV particles (Clayton et al. 2016). NiV is more likely to be transmitted from patients suffering from infection of the respiratory tract (Escaffre et al. 2013). A case-control study of risk factors for human infection with NiV during the outbreak in Malaysia showed that direct close contact with pigs was the primary source of human NiV infections, where only 8% of patients had no contact with pigs. The outbreak was stopped after pigs in the affected areas were slaughtered and proper disinfection measures were taken (Parashar et al. 2000; Chua 2010).

Changing resource landscapes, rapid change in fruit bat habitat, related shifts in their ecology and behavior, altered diet, roosting environment, movement and behaviors altogether constitute the ecological drivers causing increasing spillover risk of bat-borne viruses like *Henipavirus* to domestic animals and humans (Kessler et al. 2018). Understanding virus-bat interactions is an exciting new area of research that could through new light on the different modes regulating NiV infection and to designing effective and novel therapeutics (Enchéry and Horvat 2017).

Domesticated animals play key roles in major spill-over events of bat-borne viruses but their exact rolesas bridging or amplifying species remain unclear (Glennon et al. 2018). Their susceptibility to zoonotic viruses and potential for disease transmission to humans needs to be studied in depth in order to diminish spill-over risks of viruses like NiV and others, especially in view of global intensification of agriculture.

Spatial and temporal distribution studies of NiV spillover events in Bangladesh (2007–2013) revealed bat-to-man spillovers every winter with 36% annual variation and the distance to surveillance hospitals showed 45% of spatial heterogeneity (Cortes et al. 2018). Therefore, strategies to prevent NiV infections in humans need to be strengthened all through colder winters. Dynamics of bat infections and spillover risk need to be understood in depth, for which purpose the evolutionary studies based on codon-usage pattern can throw some lights.A recent study on the systematic evolutionary set up and codon usage pattern by both Hendra and Nipah viruses revealed that Henipaviruses are highly adapted within bats belonging to the genus *Pteropus* and this is strongly influenced by natural selection (Kumar et al. 2018).

## 4. Epidemiology and disease outbreaks

In 1998, NiV disease was recognized for the first time in Malaysia in persons who were in contact with swine population. In March 1999, one outbreak of acute Nipah virus infection was recorded in 11 male abattoir workers (average age of 44 years) in Singapore where pig meat was imported from Malaysia, with one dead. Patients showed higher level of IgM in serum and some unusual symptoms of atypical pneumonia and encephalitis with characteristic focal areas of increased signal intensity in the cortical white matter in MRI. Symptoms of hallucination along with abnormal laboratory results including low lymphocyte and platelet counts, high levels of CSF proteins and of aspartate aminostransferase were present. The patients were treated by intravenous acyclovir and eight were cured (Paton et al. 1999; Abdullah and Tan 2014). FromSeptember 1998 to June 1999, 94 patients (both males and females), with anaverage age of 37 years, reporting close contact with swine population and diagnosed with severe viral encephalitis were investigated. Results showed a direct transmission of Nipah virus from pigs to human beings. The illness showed a very short incubation period and the symptoms includedheadache, dizziness, fever, vomiting, doll’s-eye reflex, hypotonia, tachycardia, lowering of consciousness, areflexia (loss of all spinal reflexes), hypertension and high mortality (Goh et al. 2000). Surveillance studies on Malaysian wild life species like island flying foxes (*Pteropus hypomelanus*) initially revealed the seropositivity of Nipah viral antibodies in them and laterconfirmed the existence of virus also by isolation studies (Chua et al. 2002).

In Singapore and Malaysia, febrile encephalitis due to NiV has been reported from 246 patients between1998 and 1999 and in farmed pigs during the same period, as an epidemic with neurological as well as respiratory signs (CDC 1999a,b; Nor et al. 2000; Pulliam et al. 2012). Farmers associated with pig farming and abattoir workers were found to be in the high risk group (Pulliam et al. 2012), and the human mortality was about 40% (Lo and Rota, 2008). NiV infection has not been reported directly in man or pig in Indonesia, but exposure of *Pteropus vampyrus* bats to NiV has been reported. Thus in Indonesia, there is every possibility of disease spread from the carrier bats to pig or man (Woeryadi and Soeroso 1989; Mounts et al. 2001; Kari et al. 2006). Presence of anti-NiV antibodies in serum indicated an early exposure of bats to the virus. In India, a sero-surveillance study conducted over 41 pteropid fruit bats in North Indian region showed seropositivity in twenty bats (Epstein et al. 2008).

In Malaysia in 1999, human cases of Nipah viral encephalitis were initially confused with Japanese encephalitis or Hendra-like viral encephalitis. However, the Ministry of Health confirmed that NiV was the causative agent of the infection in pigs and man and morbidity was higher (231 cases out of 283 cases reported) in Negri Sembilan region of Malaysia. Genome of the NiV was sequenced at the CDC, Atlanta, Georgia, USA. The Ministry of Health declared total of 101 human deaths and approximately 900,000 pigs were culled (Uppal 2000). Researchers confirmed that Nipah infections in pigs and man that occurred in peninsular Malaysia in 1998–1999 spilled over from Chiropteran bats (Yob et al. 2001). In peninsular Malaysia, an epidemiological study was conducted for three years to assess the seroprevalence of anti-NiV antibodies and the presence of virus among *Pteropus vampyrus* and *P. hypomelanus* bats of different age groups and physiological status [involving adults, especially pregnant lactating and juvenile bats (6–24 months)]. Various risk factors for NiV infection in pteropid bats were also explored. Among the two bat species, the risk of NiV and seroprevalence were higher for *P. vampyrus* (33%) than *P. hypomelanus* (11%). NiV seroprevalence and distribution showed variation (1–20%) in the *P. hypomelanus* batsand also in between the years 2004–2006 irrespective of seasons (Rahman et al. 2013). The surveillance study was performed to assess the distribution of Henipaviruses in Southeast Asia, Australasia, Papua New Guinea, East Timor, Indonesia and neighboring countries. NiV RNA was detected in *P. vampyrus* bats of *Pteropodidae* family and non-Pteropid *Rousettus amplexicaudatus* bats from East Timor (Breed et al. 2013).

In Cambodia, NiV-specific antibodies were detected in *P. lylei* (Lyle’s flying foxes). In Indonesia, such antibodies were also detected by ELISA in the sera collected from *P. vampyrus* (Reynes et al. 2005; Sendow et al. 2006; Sarma 2017). In Thailand, regular surveillance and sero-surveillance of bat population indicated the presence of NiV RNA in the saliva and urine of bats, and IgG in the serum, suggesting long term NiV persistence in them (Wacharapluesadee et al. 2005).

In Bangladesh, outbreaks of Nipah virus were initially confirmed only by the presence of anti-NiV antibodies in serum samples. However after 2004, researchers started genetic characterization of Nipah virus by detecting viral nucleic acid (Harcourt et al. 2005).Till the year 2010, overall 9 outbreaks have been recorded in Bangladesh. Raw date palm was the source of infection of the outbreak recorded during the year 2011 (Rahman et al. 2012). Such finding is further strengthened by the fact that raw date palm consumption was common in patients with fatal infection (∼65% mortality rate) (Olson et al. 2002; Luby et al. 2006; ICDDRB 2010). Another outbreak during 2011 in a remote town named Hatibandha in the Lalmonirhat district, northern Bangladesh, reported 15 deaths due to NiV infection (Wahed et al. 2011). Studies performed in pigs in Ghana suggested that serum antibodies against Henipaviruses including Hendra and Nipah viruses and viral nucleic acid were also present in another species of fruit bat, i.e. *Eidolon helvum*, reflecting the exposure of pigs to these bats (Hayman et al. 2011).

NiV was detected for the first time in Siliguri, West Bengal, India in the year 2001 during an outbreak characterized by febrile illness in association with altered sensorium (poor thinking capability or poor concentrating capacity). A close resemblance had been found between the isolates of Siliguri outbreak and those obtained during the outbreak in Bangladesh. Such resemblance is justified, as Siliguri is located at the vicinity of Bangladesh (Harit et al. 2006; ICDDRB 2011). Another outbreak was reported from Nadia district, West Bengal in the year 2007 (Chadha et al. 2006). Most recently in the year 2018, Nipah viral disease outbreak has been reported in Kozhikode district, northern Kerala, India and the fruit bats have been identified as the source of the outbreak (Chatterjee 2018; Paul 2018). During this outbreak, deaths occurred in the infected subjects as well as in healthcare personnel who were involved in treatment of patients. On May 19, 2018, 4 infected people died and on 23 May, 2018 13 more subjects deceased (3 from Malappuram and 10 from Kozhikode district). NiV was confirmed upon laboratory testing using RT-PCR. Genetic analysis at the early stage confirmed NiV etiology and that the epidemic strain showed close resemblance to the BD strain of NiV (http://gvn.org/update-on-the-nipah-virus-outbreak-in-kerala-india/). In both outbreaks, circumstantial evidences suggested the human-to-human transmission, as most people who acquired the infection were either care-givers, or family members of infected persons.

Sporadic NiV outbreaks, person-to-person transmission and its zoonotical aspects have been implicated in hundreds of human deaths during the past two decades, and this has posed a huge threat to domestic animals and humans. Epidemiological investigations have revealed NiV circulation in Asia, Africa, and the South Pacific Ocean (Sun et al. 2018). A recent report indicated NiV outbreaks presently being still small but posing a significant threat to be extremely lethal (Spiropoulou 2018).

NiV disease outbreak investigation in Kerala, India, during May–June 2018, elucidated virus transmission dynamics and epidemiological analysis by employing real-time RT PCR testing to detect presence of virus in throat swabs, blood, urine and CSF. A total of 23 cases were identified including the index case, and 18 laboratory confirmed cases. The incubation period was recorded to be 9.5 days (6–14 days). Twenty cases (87%) showed respiratory symptoms and the case fatality rate was 91% with only two survivors. Sequencing and phylogenetic analysisrevealed NiV isolate to be closer to the Bangladesh lineage (Arunkumar et al. 2018). Nevertheless, there is a growing demand for increasing public awareness regarding the transmission pattern and risk of NiV infection which will ultimately aid in the potential reduction ofoccurrence and associated spread/outbreaks of the disease (Yu et al. 2018).

A firefly luciferase that expresses NiV has been generated for facilitating studies (spatiotemporal) on the pathogenesis of Henipaviruses. Herein bioluminescence imaging technique has been used for monitoring of the replication of the virus as well as spread in knockout mice. This reverse genetics system may be a useful tool to investigate Henipa-like viruses (Yun et al. 2015).

## 5. Phylogenetic analysis of NiV

A pair-wise-similarity analysis among the nucleotide sequences retrieved from the Nipah Virus (NiV) N and G genes was carried out after aligning the sequences by the Clustral V program in MegAlign software of the DNASTAR software package. For the genetic relatedness study, representative 1599- and 1809 bp-length for N gene (27 strains) and G gene (15 strains), respectively, were investigated. NiV strains from different countries including Malaysia, Cambodia, Bangladesh, India and Thailand, submitted during 2001–2018 were retrieved from the NCBI database. Phylogenetic analysis was performed using the maximum likelihood method (1000 bootstrap replicates) in MEGA 6 software (v 6.06) (Tamura et al. 2013). The suitable dendrogram analysis model was identified by using the find best DNA/protein model tool available in MEGA 6 (v 6.06), confirmed with the FindModel online tool (Posada and Crandall 1998). For N and G genes, the respective models were KHY + G and T92.

**Figure 3.**
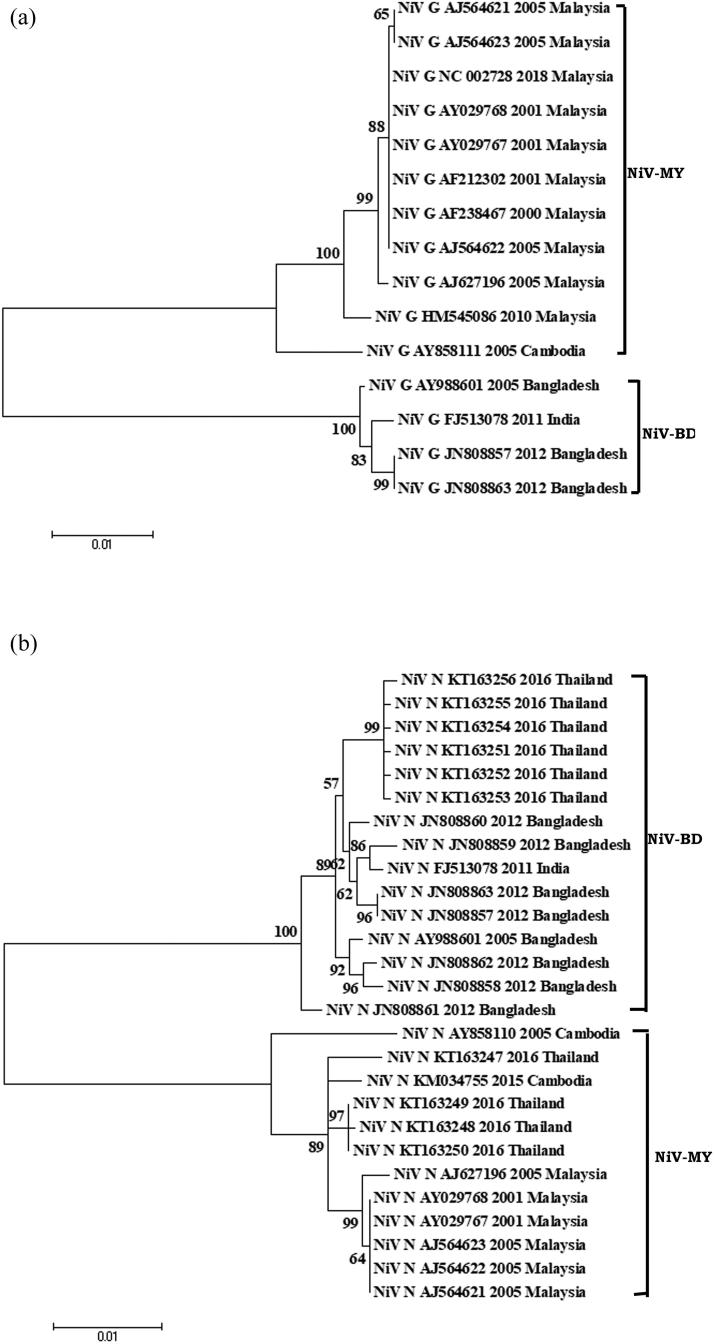
Phylogenetic analyses of sequences of Nipah Virus (NiV) strains from different countries (Bangladesh, Cambodia, India, Malaysia, and Thailand). (A) Phyloanalysis based on complete G gene (1809 bp) and (B) Phyloanalysis based on complete N gene (1599 bp). Tree created with maximum likelihood method with 1,000 bootstrap replicates. Scale bars indicate number of sequence changes corresponding to illustrated branch length. Major two NiV clades are mentioned in the side bar as BD (Bangladesh) and MY (Malaysia).

The phylogenetic analysis affirmed the circulation of two major clades of NiV, i.e. NiV-BD and NiV-MY, based on currently available complete N and G gene sequences (Figure 3A,B). The NiV isolates from Malaysia and Cambodia clustered together in NiV-MY clade, whereas isolates from Bangladesh and India clusterered within NiV-BD clade. Nonetheless, NiV isolates from Thailand harbored mixed population of sequences and distribution of the Thai isolates was seen in both NiV-MY and NiV-BD, based on N gene sequences. Notably, intra-clade (NiV-MY and NiV-BD) sequence similarity of G gene at nucleotide level was high between 98 and 100%, while inter-clade similarity was relatively less, i.e. 92.2–93.0%. Similar observation was recorded with N gene sequences where intra-nucleotide similarity in NiV-MY and NiV-BD isolates ranged between 99.1 and 100% and the inter-clade similarity was between 93.6 and 94.6%. The inference from analysis affirms circulation of two populations of NiVs currently.

## 6. Molecular epidemiology

For comparison of the open reading frame sequence of the NiV with those from other members of the *Paramyxovirinae* subfamily, phylogenetic analysis had been used widely and by such approach the closest relation between NiV and Henipavirus has been proven (Chua et al. 2000). It has been revealed by nucleotide sequencing technique that there exist very little difference in the nucleotide sequences of NiV isolated from throat secretion and cerebrospinal fluid (difference by just 4 out of 18,246 nucleotides) (Arankalle et al. 2011). Nucleotide sequence homology has also been observed between the virus isolated from Bangladesh and Malaysia but it is interesting to note that nucleotide heterogeneity (inter-strain) had been found to be more obvious. It is interesting to note that differences in genetic variability certainly have relation with the mode of transmission. It is evident by molecular epidemiological studies that NiV had been introduced in pigs in Malaysia during 1998–1999 causing great loss to pig farming (Looi and Chua 2007). However, the human and pig isolates in Malaysia during the later phase of outbreak showed nearly identical sequences. This is suggestive of the fact that there was rapid spread of only one variant in pig and such variant was responsible for most of the cases in man. In contrast, the introduction of NiV from fruit bats to humans for multiple times in Bangladesh might be responsible for the sequence heterogeneity of the NiV isolates (Chan et al. 2001; Chakraborty 2012). Detailed phylogenetic analyses have been performed on thecomplete gene sequences of NiV strains from the year 2008 as well as 2010 outbreaks in Bangladesh. On the basis of a nucleotide sequence window (comprising of 729 nucleotides), a genotyping scheme has been introduced. An accurate and simple way for classification of current as well as future sequences of NiV has been provided by this genotyping scheme. A phylogenetic tree (with very high bootstrap values) has been constructed by such genotyping method. Phylogenetic analysis showed close similarity of sequences obtained from pigs and humans during the Malaysian outbreak. Analysis also revealed that the virus isolated from Bangladesh possesses an additional 6 nucleotides than the prototype Malysian strain (Angeletti et al. 2016). For classification of sequences of NiV such methodology and phylogenetic tree is very helpful (Lo et al. 2012). For investigating the viral genetic diversity, a phylogenetic study of the infection caused by NiV has helped in estimating the infection spread and its date of origin (Lo Presti et al. 2016).

## 7. Immunobiology (immune response/immunity)

Immune response studies regarding Nipah virus have been conducted by various researchers, especially after each reported outbreak. Since the virus exhibits two dictinct types of association among its hosts (maintaining its persistence in the nature through reservoir hosts like bats and inflicting fatal clinical condition in humans as well as domestic animals like pig), the immune responses might be host-specific(Chua et al. 2000; Negrete et al. 2005; Kulkarni et al. 2013). Several proteins of Henipaviruses block host innate immune responses viz., P/phosphoprotein; V protein; the C and the W proteins. In response to several stimuli the IFNα/β production can be inhibited by V as well as W proteins whereas the ability of IFNs for signaling are blocked by P, V as well as W proteins, leading to induction of a state of cellular antiviral response (Basler 2012). The innate immune system of pteropid bats is remarkable for its constitutive action of Type 1 interferon system (which can restrict the early viral replication within their body) (Zhou et al. 2016). This mode of action has been associated also with several interferon stimulated genes (ISG) particularly of those involved in noninflammatory pathways so that elevation in interferon response in bats is not allied with chronic inflammation unlike in case of rodents or humans (Halpin et al. 2011). Due to these differences, bat cells are primed to react to viral attack immediately but only upto a level of restricting replication (Zhou et al. 2011a,b). Bats possess comparatively higher repertoire of naive immunoglobulins with more specifities, thereby favouring direct clonal selction of B lymphocytes for antibody production. In such condition there may be poor or no hypermutation and affinity maturation stages in B cells, leading to poorer responses and restricted production of high-titered antibodies than other species. These features contribute for the delay in viral clearance and persistence of virus for a pretty long period (Wellehan et al. 2009; Schountz et al. 2017). Nipah virus comparative studies conducted in pteropid bats and hamster reinforce these points as virus showed lesser multiplication and shedding from bat endothelial cells as well as with poorer antibody responses upon challenge studies (Wong et al. 2003; Lo et al. 2010; de Wit et al. 2011). Recently, tetherin (an IFN-induced protein from bats) has been reported to inhibit NiV replication in fruit bat cellsand to act as an innate immune antiviral protein that can facilitate the host to combat virus induced pathological changes (Hoffmann et al. 2018).

Another immune mechanism within bats to prevent complete elimination of Nipah virus is the modulation of bat antiviral responses towards virus survival. In the reservoir host, the virusemploys immune evasion strategies especially against innate immune system so as to escape from the immune attack and maintain perpetuation within the host by retaining replication at a minimum level (Rodriguez and Horvath 2004; Rupprecht et al. 2011). Such evasion strategies are mediated through accessory proteins encoded within the virus which may also have effect over other hosts through spillover adaptation (Schountz 2017). The NiV P gene (coding for polymerase-associated phosphoprotein) playsa key role in evading interferon mediated immune response from the host (Shaw, 2009). This gene encodes accessory proteins such as P, V, W and C; all of these were reported to inhibit host antiviral responses through blockage of interferon mediated signaling pathways, especially STAT1 stimulated JAK-STAT signaling pathway (Shaw, 2009; Prescott et al. 2012).

In case of hosts exhibiting clinical disease from Nipah virus, various virus associated immune antagonistic proteins subvert host immune responses, thus leading to pathogenesis and clinical condition. Wild type virus uses an unique RNA editing mechanism for the controlled transcription and translation of multiple antagonistic proteins which may be delayed in some hosts, so that the protein production may be slightly delayed. In such situations antiviral responses would be strong with associated inflammatory responses thus partially restricting viral replication and pathogenesis in some hosts (Seto et al. 2010). Although Nipah virus effectively suppresses antiviral cytokine production at early phase of infection, release of some amount of inflammatory cytokines has been suggested which can be attributed to the elevation in vascular permeability, ultimately favoring viral spread (Schountz 2014).

Presence of antigen-positive inclusions in the brain tissues of patients with Nipah Viral encephalitis points to the inadequacy of both innate and adaptive responses for preventing viral spread. These findings suggest the inability of dendritic cells residing at primary entry point of virus; especially respiratory tract and lungs, rendering inefficient antigen capturing and tissue restriction (Chua et al., 1999; Chong and Tan 2003).

Evidence also suggest the suppression of MHC-I expression in immune cells by the viral proteins, leading to a repression in both antigen presentation by antigen presenting cells and stimulation for mounting adaptive responses, ultimately resulting in viral spread and persistence in other target organs (Dasgupta et al. 2007; Seto et al. 2010). Besides these, the virus induced immune evasion for long time also accounts for the persistence of virus in brain tissues and ensuing relapsed and late onset fatal encephalitis in man (Tan et al. 2002). Apart from these findings, typical interaction pattern of the virus with other critical genes of the host such as TLR genes of host defence, Notch genes of neurogenesis, and other genes like TJP1, FHL1 and GRIA3 concerned with blood-brain barrier and encephalitis, etc. have been reported by computational prediction. Crucial role of miRNAs present in NiV genome in inhibiting these host genes, thereby aiding the viral spread and pathogenesis has been reported (Saini et al. 2018). The pathogenecity of Nipah virus in pigs and man can be correlated with its ability and magnitude to evade immune responses in reservoir host. Though the virus has undergone frequent species jumping involving various hosts, higher fatality rates are being associated with human outbreaks so far, which warrants a comprehensive study to elucidate and explore the viral evolution and adaptation in different hosts.

## 8. The disease

### 8.1. Pathogenesis

In the initial stage of illness in man, detection of NiV can be done in epithelial cells of the bronchiole (Chua et al. 2001). Viral antigens can be detected in bronchi and alveoli in experimental animal models; the primary targets being epithelium of bronchi and type II pneumocytes (Rockx et al. 2011). Inflammatory cytokines are induced due to infection of the epithelium of the respiratory tract; thereby recruiting cells of the immune system and ultimately leading to development of acute respiratory distress syndrome (ARDS)-like disease (Rockx et al. 2011). Significant inflammatory mediators, viz., interleukin (IL)-1α, IL-6, IL-8; granulocyte-colony stimulating factor (G-CSF), C-X-C motif chemokine 10 (CXCL10), etc. are induced when the airway epithelium (smaller ones) get infected (Escaffre et al. 2013).

From the respiratory epithelium, the virus is disseminated to the endothelial cells of the lungs in the later stage of the disease. Subsequently, the virus can gain entry into the blood stream followed by dissemination, either freely or in host leukocyte bound form. Apart from lungs, spleen and kidneys along with brain may act as target organs leading to multiple organ failure (Rockx et al. 2011; Escaffre et al. 2013). There is development of lethal infection in hamsters when leukocytes loaded with NiV are passively transferred (Mathieu et al. 2011). In pigs, there is productive infection of monocytes, natural killer (NK) cells along with CD6 + CD8+ T lymphocytes (Stachowiak and Weingartl 2012).

**Figure 4.**
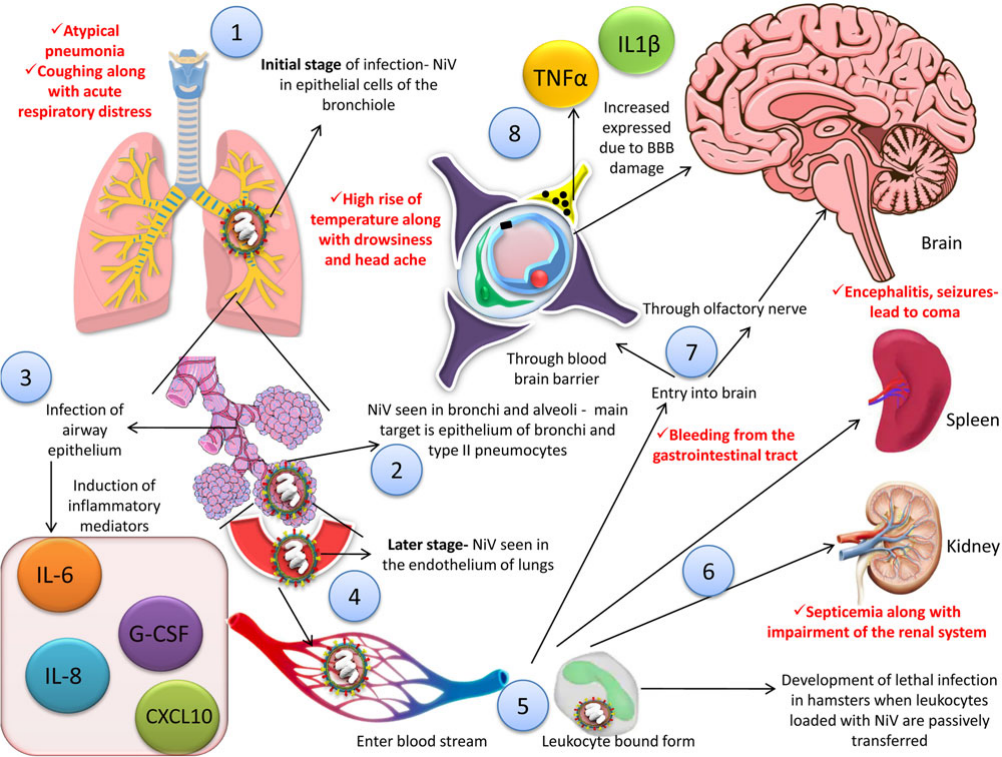
Pathogenesis of NiV. 1. NiV can be seen in the epithelial cells of the bronchiole in the initial stage of infection. 2. NiV antigen can be detected in bronchi and alveoli. 3. Inflammatory mediators are activated as a result of infection to the airway epithelium. 4. Virus is disseminated to the endothelial cells of the lungs in the later stage of the disease. 5, 6. Virus enter the blood stream followed by dissemination, either freely or in host leukocyte bound form, reach brain, spleen and kidneys. 7. Two pathways are involved in the process of viral entry into the central nervous system (CNS), *via* hematogenous route and anterogradely *via* olfactory nerve nerves. 8. The blood brain barrier (BBB) is disrupted and IL-1β along with tumor necrosis factor (TNF)-α are expressed due to infection of the CNS by the virus which ultimately leads to development of neurological signs. Red font shows the symptoms in human.

Two pathways are distinctly involved in the process of viral entry into the central nervous system (CNS), *viz*., *via* hematogenous route (through choroid plexus or blood vessels of the cerebrum) and/or anterogradely *via* olfactory nerves (Weingartl et al. 2005). The blood brain barrier (BBB) is disrupted andIL-1β along with tumor necrosis factor (TNF)-α are expressed due to infection of the CNS by the virus which ultimately leads to development of neurological signs (Rockx et al. 2011). There may be presence of inclusion bodies in case of infected CNS in man. In both the gray as well as white matter plaques may be evident along with necrosis (Escaffre et al. 2013). It is quite noteworthy that the virus can directly enter the CNS in several experimental animal models *via* the olfactory nerve. The olfactory epithelium of the nasal turbinate is infected by NiV in such animal models. The viral infection subsequently extends through the cribiform plate into the olfactory bulb. Ultimately, the virus is disseminated throughout the ventral cortex along with olfactory tubercle (Weingartl et al. 2005; Munster et al. 2012; Escaffre et al. 2013). A diagrammatic representation of pathogenesis of NiV has been depicted in Figure 4.

### 8.2. Clinical signs and symptoms

Highly pathogenic NiV causes symptomatic infections in pigs and humans. Respiratory symptoms are much more severe in pigs as compared to humans.

#### 8.2.1. In humans

The virus is responsible for causing severe and rapidly progressing illness in humans with the respiratory system as well as the central nervous system (CNS) mainly getting affected (Hossain et al. 2008). The signs and symptoms of the disease appear 3–14 days post NiV exposure. Initially, there is a high rise of temperature along with drowsiness and headache. This is followed by mental confusion as well as disorientation, ultimately progressing towards coma within 1–2 days. A critical complication of the NiV infection is encephalitis. During initial phase, the respiratory problems may become evident. There is development of atypical pneumonia. Coughing along with acute respiratory distress may be evident in certain patients (Hossain et al. 2008; Williamson and Torres-Velez 2010). There may be sore throat, vomiting, along with muscle aches (www.medicinenet.com). There may be development of septicemia along with impairment of the renal system and bleeding from the gastrointestinal tract. In severe cases within a period of 24–48 h, there may be development of encephalitis along with seizures that ultimately leads to coma (Giangaspero 2013). It is crucial to note that transmission of the virus is more common from patients having labored breathing than those having no respiratory problems (www.cdc.gov; Luby et al. 2009).

#### 8.2.2. In animals

In pigs, the disease is also known as porcine respiratory and encephalitis syndrome (PRES), barking pig syndrome (BPS) (in peninsular Malaysia) or one-mile cough. An acute febrile illness has been reported in pigs below six months of age wherein there is development of respiratory illness that ranges from rapid labored breathing to non-productive cough which is harsh in nature. With the exception of young piglets, the mortality is relatively low (Nor et al. 2000; Chua 2003; Giangaspero 2013). In animals that are confined, morbidity may approach 100 per cent (Nor et al. 2000). Due to involvement of nervous system, there may be twitching of muscles, weakness of hind legs, tremors, along with paresis, either flaccid or spastic, of varying degrees. There may also be nystagmus along with seizures in boars as well as sows (Chua 2003; Kulkarni et al. 2013). In dogs infected with NiV, there may be inflammation of the lungs along with necrosis of glomeruli as well as tubules with formation of syncytia in kidneys. In cats, there may be development of endothelial syncytia along with vasculopathy in multiple organs. Experimental NiV infection of various animals, *viz*., hamster, guinea-pig, chick embryo, as well as African green monkey, results in development of lesions in the parenchyma in the CNS along with vasculopathy. Clinical signs are, however, apparently absent in mice as well as rats for unknown reasons (Wong and Ong 2011; Kulkarni et al. 2013).

### 8.3. Postmortem findings

Magnetic resonance imaging (MRI) studies in human patients have revealed that the cortex, pons as well as temporal lobes of brain get involved. There may be bilateral abnormalities in the white matter of the brain. In the cerebral cortex, there may be more than one hyperintensities (T1-weighted) which are very much similar to necrosis of the laminar cortex. Lesions may also become evident in corpus callosum, brain stem, as well as cortex of the cerebrum. It is crucial to note in this regard that diffusion-weighted (DW)-MRI is employed to detect such lesions (Goh et al. 2000; Lim et al. 2002; Ang et al. 2018). There may be presence of disseminated microinfarction in the brain due to thrombosis induced by vasculitis. The neurons may also get involved directly. Vasculitic lesions of similar nature may be found in the kidneys, heart, as well as respiratory tract (Ang et al. 2018). It is also interesting to note that blood vessels of medium and small size show most involvement in case of infection due to NiV, which results in development of syncytia (multinucleated) along with fibrinoid necrosis (Ang et al. 2018).

There may be consolidation of varying degree along with hemorrhages (either petechiae or ecchymosis) in the lungs of affected pigs at necropsy. Froth-filled bronchi along with trachea are commonly observed. In certain instances, there may be presence of blood stained fluids in the trachea and bronchi. Congestion along with generalized edema is present in kidneys and brain. Both the cortex as well as suface of kidneys may become congested (Nor et al. 2000). There may be pneumonia (moderate to high) along with formation of syncytial cells in the endothelial cell lining of the blood vasculatures as revealed histologically (Chua et al. 2000; Nor et al. 2000). In the CNS and other major organs like lungs and kidneys, there may be development of small vessel vasculopathy (disseminated) in case of acute infection (Wong et al. 2009). Generalized vasculitis along with fibrinoid necrosis and mononuclear cell infiltration may be noticed in the brain, kidneys and lungs. Viral antigens at greater concentration may be present in the blood vascular endothelial cells (especially in the lungs) as is revealed immunohistologically. In the upper respiratory tract of pigs in the lumen viral antigens are evident amidst the cellular debris which is suggestive of the possible transmission of NiV through exhalation (Nor et al. 2000; Kulkarni et al. 2013). In dogs, kidneys may show congestion with severe hemorrhage. Exudates may be present in the bronchi and trachea (Nor 1999; Kulkarni et al. 2013).

## 9. Public health significance and zoonotic aspects

NiV is the most recently emerging zoonotic and highly deadly virus having pandemic threat. As an emerging and recognized zoonotic pathogen discovered in modern times, NiV causes severe febrile illness and high fatality rates in affected persons and is posing an ongoing high risk to the health of humans worldwide (Clayton 2017; Mukherjee 2017; Thibault et al. 2017). NiV is an uncommon but has become a deadly virus responsible for causing high fatality rates of 40–75%. Fruit bats (*Pteropus*) serve as natural hosts (wildlife reservoir) and pigs are the intermediate hosts for NiV zoonotic cycle (Paul 2018). During a large outbreak of acute encephalitis in Malaysia in 1998, the virus was discovered in affected patients having contact with sick pigs. The pigs got infection from bats, and then NiV spread proficiently among pig-to-pig, and thereafter from pig-to-man. Moreover, it has been revealed that *Pteropus vampyrus* and *Pteropus hypomelanus* (flying foxes in the Malysian Islands) bear the virus in saliva as well as urine, indicating their potential to act as natural reservoir of the virus (Looi and Chua 2007). It is interesting to note that there is always risk of spill over associated with NiV infection. Interaction of the molecular as well as ecological factors collectively that govern the susceptible nature of populations of animals (domestic) as well as humans are not understood yet well (Thibault et al. 2017).

Besides Malaysia, the fruit bats of *Pteropus* genus serve as the main reservoir of NiV in Thailand and Cambodia. Apart from drinking raw date palm sap contaminated by bats as a cause of initial outbreak, man-to-man and animal-to-man transmission is also a major mode of spread of the infection during an ongoing outbreak. Further, it has been found that direct contact of the susceptible population with the respiratory and body secretions of the infected patients increases the risk of acquiring the infection. During the NiV outbreak in Thakurgaon district, northwest Bangladesh, anti-NiV antibodies were detected in half of the *Pteropus* bats tested (Chadha et al. 2006; Gurley et al. 2007; Homaira et al. 2010a,b; Clayton 2017; Thibault et al. 2017). Other major public health threats appear to be acquiring NiV infection from the susceptible food and domestic animals. Many domesticated mammals seem to be susceptible to Nipah virus. This virus can be maintained in pig populations, but other domesticated animals such as sheep, goats, dogs, cats and horses appear to be incidental hosts acquiring the infection during outbreaks. Fruits punctured by the bat and contaminated with their saliva forma common source of transmission of NiV infection from bats to domestic animals. Consumption of fruits eaten partially by fruit bats may cause infection in pigs which may then transmit it to humans. Contact with sick cow was reported to have caused a case of human infection in Bangladesh (Chua 2003; Luby et al. 2012; Siddique et al. 2016; http://www.cfsph.iastate.edu/Factsheets/pdfs/nipah.pdf).

Consumption of fruits, vegetables or water contaminated with saliva, urine or fecal matter of infected bats could also be a possible mode of transmission to man and animals (Luby et al. 2009). Date palm sap can be used to prepare alcoholic beverages and such beverages when consumed can lead to human infection (Harit et al. 2006; Simons et al. 2014). Evidence from several NiV outbreaks indicate that consumption of undercooked meat from infected animals or handling of infected animals in the home, farm or slaughter houses may also pose risk of animal-to-man transmission (Chanchal et al. 2018).

Close contact with symptomatic patients or their infectious secretions has been implicated for human transmission of NiV in Bangladesh. Specific exposures can pose a high risk of person-to-person transmission, though sustained transmission do not occur in humans. Studies conducted in animal models further support this fact (Clayton 2017). The recent NiV outbreak in Kerala, India, which caused encephalitis in humans, raised global health concerns (Paul 2018).

The potential for a global pandemic due to NiV appears to stem from several features: availability of susceptible human population, several viral strains withpotential for person-to-person transmission, and error-prone nature of RNA virus replication. Outbreaks of NiV disease in densely populated regions like South Asia can lead to pandemics, due to extensive global travel and trade connectivity (Luby 2013). Many ecological and molecular factors underlie NiV spillover into humans and human and animal susceptibility to it, though the intricate interaction between these is unclear (Thibault et al. 2017). Research studies need to be undertaken to elaborate the molecular mechanisms of the respiratory transmission of NiV in order to reduce the risk of human-to-human transmission. Improved surveillance and vaccination strategies must also be adopted (Luby 2013).

## 10. Laboratory diagnosis

Confirmation of the human as well as animal NiV infections can be done by isolation of the virus along with performing serological tests and tests to amplify viral nucleic acids. Biosafety level-4 (BSL-4) laboratory facilities are required for NiV isolation as well as propagation. However, BSL-3 may prove to be sufficient to primarily isolate the virus from suspected clinical materials. Following confirmation of the virus in infected cells (fixed by acetone) by immunofluorescent technique, there should be immediate transfer of the culture fluid in BSL-4 laboratory (Daniels et al. 2001; Ksiazek et al. 2011). It is crucial to note in this aspect that International Centre for Diarrhoeal Disease Research, Bangladesh (ICDDRB) along with Institute of Epidemiology Disease Control and Research (IECDR) are the institutes involved in handling NiV in Bangladesh. In India, BSL-4 laboratory has been established in Pune at National Institute of Virology (NIV) (Kulkarni et al. 2013). In Japan, National Institute of Animal Health has developed immunohistochemical diagnostic technique based on monoclonal antibodies (Tanimura et al. 2004).

Viral antigen capture ELISAs offer a high-throughput and inexpensive method for screening suspect samples. A monoclonal antibody-based antigen capture ELISA has been reported for detecting NiV as well as to differentiate it from HeV (Chiang et al. 2010). Indirect IgG ELISAs have been developed for testing swine and human sera, and an IgM capture ELISA using a recombinant N protein of NIV has also been reported (Yu et al. 2006). A sandwich ELISA employing a rabbit polyclonal anti-NiV G protein has been reported and represents a quick test for diagnosing the disease (Kaku et al. 2012). Infected cell lysate containing NiV antigens can be used as coating agent for conducting ELISA. Australian Animal Health Laboratory (AAHL) (Geelong) and Centers for Disease Control and Prevention in Atlanta provide such antigens (Eshaghi et al. 2004; Chen et al. 2006; Yu et al. 2006).

In order to screen the serum samples of pigs, a recombinant N protein based-ELISA has been developed at the High Security Animal Disease Laboratory (HSADL), Bhopal. By the use of pseudotyped particles, a serum neutralization test for NiV can be performed under BSL-2 conditions. This test uses a recombinant vesicular stomatitis virus that expresses secreted alkaline phosphatase (SEAP). Neutralization titer can be obtained by measurement of SEAP activity (Kaku et al. 2012). Microsphere assay (luminex based) has been used for detection of antibodies against a glycoprotein of NiV, namely NiV sG, in the sera of pigs and ruminants like goats and cattle (Chowdhury et al. 2014). Recently, ELISA has also been developed using recombinant full length N protein and truncated G protein for detecting virus specific antibodies in serum samples of porcines (Fischer et al. 2018). NiV N ELISA was employed for initial screening of serum samples for henipavirus infection, while NiV G ELISA detected specifically the NiV infections. Such ELISAs are valuable diagnostic methods for seromonitoring of swine population and probably livestock and wildlife animals.

Molecular tests such as reverse transcription polymerase chain reaction (RT-PCR) along with real-time RT-PCR (qRT-PCR) and duplex nested RT-PCR (nRT-PCR) have been found useful for detection of NiV infection, with subsequent confirmation by nucleotide sequencing of amplicons. A unique primer set targeting the N gene has been reported. Internal controls may also beincluded in nRT-PCR tests for detection of NiV RNA. Further, such kind of nRT-PCR has helped to detect two different viral strains from *Pteropus lylei* in Thailand (Chua et al. 2000; Guillaume et al. 2004a; Wacharapluesadee and Hemachudha 2007). qRT- PCR protocols have also been developed fordetection of henipaviruses and found to be useful for the diagnosis of NiV infection as well (Wang and Daniels 2012; Kulkarni et al. 2013; Jensen et al. 2018). SYBR-Green I dye-basedqRT-PCR employing primers specific to N gene have also been reported (Chang et al. 2006). Recently, a novel one step qRT-PCR assay targeting the intergenic region separating F and G genes has been reported for quantitative detection of NiV replicative viral RNA that avoids viral mRNA amplification, and may represent a more precise assay than the conventional qRT-PCR (Jensen et al. 2018). Advancements in the field of diagnosis of emerging zoonotic pathogens following an integrated One Health approach need to be explored optimally (Bird and Mazet 2018).

## 11. Vaccines

Vaccination of humans is an integral part of preventing infection due to NiV. Prevention also includes vaccination of livestock (especially pigs and probably horses) in endemic areas (Broder et al. 2016). Of note, outbreaks cannot be prevented amongst the livestock population in areas where contamination of date palm sap acts as major contributor to the spread of NiV infection. However, if vaccination of livestock is made cheap it may prove to be successful in certain regions. Extensive research involving preclinical studies in a number of animals and nonhuman primates have identified multiple vaccine candidates, including vectored and subunit vaccines, offering protective immunity (Satterfield et al. 2016a). Among vectored vaccines, one employing vesicular stomatitis virus has shown protection inferrets, African green monkeys, as well as hamsters (Mire et al. 2013). Despite these developments, funding for human clinical trials of candidate vaccines remains a problem for academic community.The pharmaceutical companies are hesitant to invest in research on development of vaccines for diseases like Nipah, which are rare occurrences, despite the high fatality.

A collaborative effort has been undertaken by both government and pharmaceutical companies, known as the Coalition for Epidemic Preparedness Innovations (CEPI). It was formed in January 2017 for developing safe, efficacious and affordable vaccines against diseases associated with pandemic potential, like Nipah (Satterfield 2017). NiV, Lassa virus and Middle East Respiratory Syndrome Coronavirus (MERS-CoV) have been afforded high priority by CEPI. CEPI aims to develop two new experimental vaccines within five years, in the first phase of the clinical trial. It is anticipated that field efficacy studies of such vaccines could be done during massive outbreaks (Satterfield 2017). CEPI has recently signeda $25 million contract with two US Biotech companies, i.e. Profectus BioSciences and Emergent BioSolutions, to accelerate the work on developing a vaccine against the NiV.

DNA vaccines, virus-like particles, virus vectors(live and recombinant), and other advanced vaccines have been developed as strategies of immunization against both HeV and NiV (Walpita et al. 2011; Kong et al. 2012; Kurup et al. 2015). Experimental vaccines based on the several viral vectors, including the canarypox virus, vesicular stomatitis virus glycoprotein (VSVΔG) and rhabdovirus have been evaluated (Weingartl et al. 2006; Chattopadhyay and Rose 2011; Lo et al. 2014; Kurup et al. 2015; Satterfield et al. 2016a).

A recombinant measles virus (rMV) vaccine that expresses envelope glycoprotein of NiV has been found to be promisingfor use in man (Yoneda 2014). A replication-competent, recombinant VSV-vectored vaccine encoding NiV glycoprotein was reported to show high efficiency in a hamster model. A single intramuscular dose of the vaccine conferred protective immunity in African green monkeys one month after vaccination (Prescott et al. 2015). Healthcare workers and family contacts attending Nipah cases should be considered for Nipah vaccination, in order to limit human-to-human transmission and curb outbreaks (DeBuysscher et al. 2016). A very strong virus-specific immune response is generated through vaccination which inhibits the virus replication and shedding. Such vaccine could provide protection from NiV in disease outbreaks. Attenuated live vaccines as well as subunit G (recombinant platforms) have also been tested (Satterfield et al. 2016a).

Nipah virus-like particles (NiV-VLPs) composed of three NiV proteins G, F and M derived from mammalian cells have been produced and validated as vaccine in BALB/c mice. The immunogenicity of the NiV-VLP vaccine was high because the VLPs possess the native characteristics of the virus including the size, morphology and surface composition (Jegerlehner et al. 2002; Jennings & Bachmann 2007; Walpita et al. 2011; Liu et al. 2013). A recent work reported a novel strategy of adding a cholesterol group to the C-terminal heptad repeat (HRC) of the F protein that facilitated membrane targeting and fusion of the peptide. Enhanced penetration of the central nervous system and significant increase in antiviral effects were observed with these peptides (Porotto et al. 2010). NiV-VLPs derived from mammalian cells transfected with plasmids containing NiV G, F and M genes have also been produced yielding VLPs with the three proteins. These VLPs are composed of G, M and F proteins of the virus. Golden Syrian hamsters immunized with these VLPs developed high titres of neutralizing antibody in serum, and showed complete protection upon viral challenge (Walpita et al. 2017).

Immunoinformatic advances have been utilized for developing peptide-based NiV vaccine by prediction and modeling of T-cell epitopes of NiV antigenic proteins. Specific epitopes, *viz*., VPATNSPEL, NPTAVPFTL and LLFVFGPNL of N, V and F proteins, respectively, showed substantial binding energy as well as score with HLA-B7, HLA-B*2705 and HLA-A2 MHC class-I alleles, respectively (Kamthania and Sharma 2015). Such predicted peptides can potentially stimulate T-cell-mediated immunity and could have utility in developing epitope-based vaccines to counter NiV. *In silico* epitope prediction tools which evaluated G and F protein of NiV indicated that either GPKVSLIDTSSTITI or EWISIVPNFILVRNT peptides could formulate an effective universal vaccine component, inducing both humoral and cell-mediated immunity (Sakib et al. 2014). A more recent *in silico* analysis using bioinformatics tools indicated that the epitopes from G (VDPLRVQWRNNSVIS) and M (GKLEFRRNNAIAFKG) proteins can be helpful for designing common B- and T-cell epitope-based peptide vaccinesagainst HeV and NiV, and this approach needs to be evaluated (Saha et al. 2017). From another epitope-based immunoinformatics and prediction study on the NiV associated RNA-dependent RNA polymerase protein complex, best-predicted T-cell epitopes identified are ‘ELRSELIGY’ (peptide of phosphoprotein) and ‘YPLLWSFAM’ (nucleocapsid protein). Such approach identified B-cell epitope sequences in phosphoprotein (421 to 471), polymerase enzyme gene (606 to 640) and nucleocapsid protein (496 to 517). These studies are oriented for the validation of potential vaccine candidate protein portions from Nipah virus which could then spearhead towards the development of fruitful subunit vaccines (Ravichandran et al. 2018).

The development of animal models of NiV disease is another priority, in order to evaluate the preventive and therapeutic approaches. This will help in employing successful immunization strategies (both active as well as passive) by targeting the envelope glycoprotein of the virus (Broder et al. 2012).

**Figure 5.**
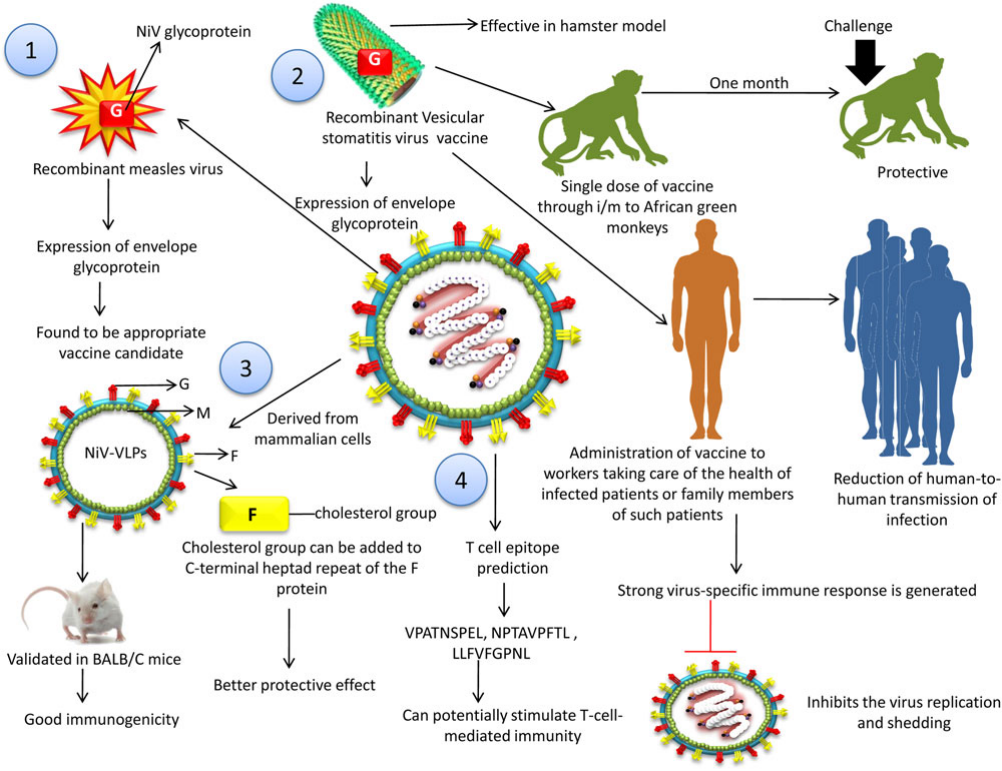
Vaccine platforms for NiV. 1. Recombinant measles virus (rMV) vaccine that expresses envelope glycoprotein of NiV has been found to be effective vaccine candidate. 2. A recombinant vaccine based on vesicular stomatitis virus (replication-competent) has been developed in recent years encoding a glycoprotein of NiV. 3. Nipah virus-like particles (NiV-VLPs) composed of three NiV proteins G, F and M derived from mammalian cells have been produced and validated as vaccine in BALB/c mice. 4. Immunoinformatic advances have been utilized for developing peptide-based NiV vaccine by prediction and modeling of T-cell epitopes of NiV antigenic proteins.

An overview on different vaccine strategies available for Nipah virus (NiV) is presented in Table 1 and few important vaccine platforms are depicted in Figure 5.

**Table 1.** Different vaccine strategies available for Nipah virus (NiV).

Vector	Antigen used	Dose for immunization	Animal model	Route of vaccination	Administration frequency	Challenge with virus titer	Route of challenge	References
Vesicular stomatitis virus (VSV)	rVSV expressing NiV G	10^5^ plaque forming units (PFU)	Hamsters	Intraperitoneal	Single	6.8 × 10^4^ TCID_50_ (1000 LD_50_)	Intraperitoneal	DeBuysscher et al. 2014
	rVSV-ZEBOV-GP-NiVG	10^7^ PFU	African Green monkey	intramuscular	Single	10^5^ TCID_50_ (Malaysian strain)	Intratracheal	Prescott et al. 2015
	rVSV-ΔG-NiVB/G	10^7^ PFU	Ferrets	Intramuscular	Single	∼5 × 10^5^ PFU	Equally divided between intratracheal and intranasal routes	Mire et al. 2013
	Replication-defective VSV	10^6^ infectious particles	Female Syrian golden hamsters	Intramuscular	Single	10^5^ TCID_50_ per hamster (>1000 times LD_50_- Malaysian strain)	Intraperitoneal	Lo et al. 2014
Canarypox virus (ALVAC) vaccine vector	vCP2199, carrying the NiV-G and vCP2208, carrying the NiV F	10^8^ PFU	Landrace female pigs	Intramuscular	Boosted 14 days postvaccination	2.5 × 10^5^ PFU	Intranasal challenge	Weingartl et al. 2006
Adeno-associated virus (AAV)	NiV G	2.1010/1.1010 genome particles	Balb/c male mice	Intra muscular or intra-dermal	One Booster	10^4^ PFU	Intraperitoneal	Ploquin et al. 2013
		6.1011 genome particles	Golden hamsters	Intra muscular				
Vaccinia virus	NiV G and NiV F	10^7 ^PFU	BALB/c female mice	Subcutaneously	Boosted with the same dose	1,000 PFU	Intraperitoneal	Guillaume et al. 2004b
Measles virus based-vectors (HL strain or Edmonston B strain)	NiV G	1 × 10^5^ TCID50	African green monkeys	Subcutaneously	One booster	1 × 10^6 ^TCID50	Intraperitoneal	Yoneda et al. 2013
		2 × 10^4^ TCID50	Hamsters	Intraperitoneal		1 × 10^8^ TCID50	Intranasal	
Venezuelan equine encephalitis virus replicon particles	NiV G	3.1 × 10^5^ IU	C3H/He mice	Foot pad inoculation	Single	Serum neutralization assay with pNL4-3.luc.E-R-reporter-gene encoding retroviruses pseudo-typed with NiV F + NiV G glycoproteins	–	Defang et al. 2010
Newcastle disease virus (NDV), LaSota strains	NiVG and NiVF	108 EID50	Mice	Intramuscular	Single	Serum neutralization assay with VSVDGnGFP-NiV G/F pseudo- typed virus	–	Kong et al. 2012
		2 × 109 EID50	Pig	Intramuscular				
Nipah virus-like particles (NiV-VLPs)	NiV G, F and M adjuvanted with Alum (50 µg -Alhydrogel 2%); MPLA (15 µg) and CpG ODN (40 µg)	30 µg VLP	Golden Syrian hamsters	Intramuscular	Three-dose vaccination schedule	∼16,000 PFU	Intraperitoneal	Walpita et al. 2017

## 12. Prevention and control measures

Outbreaks of NiV invoke costly emergency responses around the world. NiV poses a greater threat in regions associated with risk factors and with poor indicators of development (Tekola et al. 2017). With climate change and human encroachment into flying fox habitats, it is likely that outbreaks will occur in new locations (Satterfield 2017). Strategies other than vaccination also play crucial roles in prevention and control of human NiV infection and would prove to be more economical. Prevention of infection in livestock could be an efficacious strategy in regions where theyserve as intermediate hosts. It involves keeping fruit as well as bat roosting trees away from the livestock farms and grazing lands susceptible to virus contamination. In certain countries, like Malaysia, such effort is already proven to be highly effective (Satterfield 2017). The control is much tougher in regions where contaminated date palm sap is the primary source of NiV. In such regions, human behavioral changes, *viz*., drinking of contaminated date palm sap, would be necessary. In Bangladesh, date palm sap is usually harvested overnight. The nocturnal activities of bats such as drinking from, defecating or urinating in the date palm sap collection jars have become evident through infrared cameras. Measures to prevent the access of bat to the sap stream of the date palm tree as well as shaved surface can minimize the risk of human exposure to NiV in such settings (Khan et al. 2010). Creating public awareness about avoidance of consumption of raw sap and preventing contamination of the sap collection potswere the essential approaches to prevent the disease in Bangladesh during 2012–2014 (Nahar et al. 2017). Proper washing of the vegetables as well as fruits is essential to remove traces of bat excreta (https://www.ndtv.com/health/nipah-virus-some-preventive-measures-for-nipah-virus-1855891).

When the genomes of Squirrel monkey, Cynomolgus macaques and African green monkey are analyzed it sheds light on protection against the infection caused by NiV with the aid of immune factors of man. Cynomolgus macaques do not develop signs of NiV infection while Squirrel monkeys and African green monkeys develop symptomatic infection. NiV is endemic in Southeast Asia where co-evolution of the virus has taken place likely with other Henipaviruses apart from NiV. Squirrel monkeys are found in central as well as south America while African green monkeys inhabit Africa. NiV is not reported in any of these regions hence theSquirrel monkeys and African green monkeysarenaïve to NiV. Full genome sequencing along with annotation of such species of primates is possible due to the availability of improved DNA sequencing technology. This can yield insights on the host genetics conferring susceptibility of certain primate species to NiV infection and might inform on therapeutic and preventive targets in humans (Satterfield 2017).

The National Centre for Disease Control (NCDC) reported that implementation of infection control and precaution at both household and hospital levels helps to limit the NiV disease outbreak. Active surveillance and contact tracing are important along with quarantine of health professionals and peopleat high risk (http://www.ncdc.gov.in/showfile.php/lid=241). Sporadic nature of NiV outbreaks, lack of information of exact correlates of protective immunity, lack of interest among private pharmaceutical companies, and inadequate availability of BSL4 laboratory facilities to test the vaccines or therapeutics against NiV in preclinical models pose challenges to NiV vaccine development. Development of diagnostics suitable for field conditions, immunotherapeutic approaches, vaccines and antiviral drugs are urgent priorities for long-term measures aimed at prevention and control of NiV disease. Utmost care should be taken in order to avoid direct contact with the persons infected by NiV. Personal protection devices, viz., masks, glasses and gloves, should be used properly. Hydration of the patient is also important (https://www.ndtv.com/health/nipah-virus-some-preventive-measures-for-nipah-virus-1855891). In nations having no past history of Nipah viral outbreak, anticipatory preparedness for rural as well as urban outbreaks of the disease will ultimately help in prevention and control of potential outbreaks (Donaldson and Lucey 2018). Control measures adopted during the NiV outbreaks in Malaysia have shown the involvement of multidisciplinary, multi-ministerial teams in a close collaborative and cooperative manner with various agencies at international level (Chua 2010). Such approach needs to be adopted in case of the unpredictable disease outbreaks and deadly epidemics for controlling the spread of the virus (Kumar and Anoop Kumar 2018).

With recent outbreaks of emerging and re-emerging pathogens posing challenges to humanity, a rapid detection and characterization of infectious agents is being emphasized worldwide as well as laboratories are getting prepared for performing more advanced and sophisticated research to counter disease outbreaks owing to lethal pathogens flaring up in the 21^st^ Century. For this purpose, biosafety and biosecurity measures need to be specially focused so as to also prevent any accidental and/or deliberate discharge of high risk pathogens especially with zoonotic potential. Many microorganisms studied in veterinary laboratories also have the potential to infect humans (Brass et al. 2017).

Bat-borne viruses are posing high risks to human and animal health, and the present scenario demands a ‘One Health’ approach to comprehend their frequently complex spill-over routes (Glennon et al. 2018). There is an urgent need to create multidisciplinary teams as far as ‘One Health’ approach is concerned. Such team should include: medical doctors, veterinarians and agriculturists; officers from public health sectors; vector biologists as well as ecologists and phylogeneticists who can altogether put combined effort for preventing any major outbreak (Zumla et al. 2016).

The role of bats in transmission and spread of the pathogens need to be understood in depth so as to avoid cross-species spill over especially of the deadly virusesat wild and domestic animals as well as human interface. As an illustration, screening of fecal samples of bats in caves often visited by local residents to gather manure or for hunting in Zimbabwe revealed the significance of virus monitoring and surveillance in bats at sites with high zoonotic diseases transmission ability and to strengthen appropriate prevention and control measures to curtail and check the dissemination of virus to other places (Bourgarel et al. 2018).

A detailed understanding of the biogeography of the disease is required to comprehend the potential distribution of the NiV disease. Deka and Morshed (2018) carried out a study implementing certain means of modelling the risk of regional disease transmission *viz*., ENMeval and BIOMOD2. Such approaches help in measuring niche similarity between the ecological features and the *Pteropus* bats (as reservoirs of NiV). A recent bibliometric study identified a sudden increase in the number of publications referring to the eight pathogens of global concern identified by WHO, viz., Lassa, Rift Valley, Marburg, Ebola, Middle Eastern Respiratory Syndrome, Severe Acute Respiratory Syndrome, and Crimean-Congo Hemorrhagic Fever viruses (Sweileh 2017). Almost two decades after the first report of NiV, a fruitful development in the therapeutical and preventive aspect of this deadly disease is still lacking which adds to the public health threat out of it. According to the reports from the Centers for Disease Control and Prevention (CDC), several developing as well as economically deprived countries are at high risk of Nipah outbreak (Ramphul et al. 2018).

## 13. Therapeutics and treatment modalities

The essence of treatment modalities along with effective therapeutics is understood, once there is an outbreak of an infectious disease. There is a need for administering therapeutics to manage the patients during NiV outbreaks and to prevent the mortality. No specific drug has been yet approved for the treatment of this important disease. Limited work has been done to develop therapeutics against NiV infection. In preclinical studies, monoclonal antibodies have been used for treatment purposes. Due to the expensive nature of the drugs based on antibodies, identification of broad spectrum antivirals is essential along with focusing on small interfering RNAs (siRNAs) (Satterfield 2017). In animal models, the NiV pathogenesis has been understood by shedding light on the crucial nature of phospho-matrix as well as accessory proteins. For the development of novel anti-NiV drugs, such viral proteins, fusion protein and glycoprotein of the virion surface are attractive targets (Mathieu et al. 2012; Satterfield et al. 2015, 2016b; Watkinson and Lee 2016; Satterfield 2017). A monoclonal antibody targeting the viral G glycoprotein has been shown beneficial in a ferret model of the NiV disease (Bossart et al. 2009). A successful outcome of an *in vivo* study using an investigational therapeutic, i.e. fully humanized monoclonal antibody m102.4 against NiV, in a nonhuman primate model highlights the availability of potential drug for NiV treatment in future (Geisbert et al. 2014). All the 12 African green monkeys that received m102.4 survived the NiV infection, whereas the untreated control subjects succumbed to disease between days 8 and 10 after infection. It has been noticed in the recent outbreak in Kerala in South India that the antiviral drug ribavirincould be explored as anti-NiV agent (https://indianexpress.com/article/india/nipah-virus-outbreak-in-kerala-everything-you-need-to-know-5194341/). Supportive therapies such as hydration and ventilator support constitute important aspects of clinical management of NiV cases.

Properties like virulence, cell tropism, viral entry into the host cell (that includes virus attachment and receptor identification and the process of fusion of membranes of the virus and host cell), etc. have been studied extensively to develop therapeutics effective against infection caused by Henipavirus including NiV (Eaton et al. 2005; Bossart and Broder 2006). The G as well as F proteins of NiV (as well as HeV) can be targeted for inhibition of the viral entry into the host cell (Steffen et al. 2012). For treating infections caused by Henipaviruses, there were no efficacious therapeutic or prophylactic measures as is evident from the report of Vigant and Lee (2011). Even though in case of NiV outbreaks the empirical use of ribavirin has been proven to be beneficial, but its use has got dispute due to its inefficacy in case of infection caused by other Henipaviruses in various animal models (Vigant and Lee 2011).

In animal models, the recent therapeutic approaches against NiV have been validated targeting the early steps in the infection caused by the virus. These include: use of the virus neutralizing antibodies and blocking the fusion of membrane with peptides binding the fusion protein of the virus (Mathieu and Horvat 2015). Full protection has been provided by the drug favipiravir (T-705) when used for 2 weeks (either orally two times a day or through subcutaneous route once a day) in Syrian hamsters challenged with Nipah viral lethal dose (Dawes et al. 2018). The use of monoclonal antibodies, immunomodulators, convalescent plasma along with intensive supportive care are in vogue for treating severe complications associated with respiratory and nervous system (Chattu et al. 2018). Experiments have been conducted to develop concept of prophylactic use of antifusion lipopeptides against the lethal NiV. As far as developing effective lipopeptide inhibitors (with convincing biodistribution as well as pharmacokinetic features) and efficacious delivery method are concerned results of such experiments are very much crucial (Mathieu et al. 2018). Nevertheless, the pathogenesis of the Henipavirus infection including NiV should be understood in a better way for advancing the field of therapy against such kind of viral infection further (Mathieu and Horvat 2015).

Monoclonal antibodies of mouse origin or polyclonal antiserum which is glycoproteins G or F specific are used for passive immunotherapy in hamster modelwhich is found to be protective in nature (Guillaume et al. 2004b, 2006, 2009).

It is to be noted that when neurological as well as respiratory troubles prevail use of antiviral drug *viz.*, ribavirin along with intensive support care as well as immunomodulators are effective. However, the surveillance system concerning the animal health must be strengthened through a ‘One Health’ approach so that the public health authorities can be warned at an early stage (Dhama et al. 2013a; Chattu et al. 2018).

Therapeutic applications of cytokines (Dhama et al. 2013b), recombinant proteins, RNA interference technology, Toll like receptors (TLRs) (Malik et al. 2013), avian egg yolk (IgY) antibodies, plant-based pharmaceuticals (Arntzen 2015; Streatfield et al. 2015), nanomedicines (Prasad et al. 2018); immunomodulatory agents, probiotics, herbs/plant extracts (Dhama et al. 2013c, 2018a), and others may be explored appropriately to combat NiV, as these have been found promising against other viral pathogens (Dhama et al. 2013d, 2014, 2018b, 2018c; Munjal et al. 2017; Singh et al. 2017). Global adequacy of current and advanced approaches in designing efficient diagnostics, vaccine and drugs as well as their timely availability will give a high strength to counter emerging and re-emerging pathogens as well as alleviate their zoonotic impact and pandemic threats.

## 14. Conclusion and future directions

Over the past two decades, the Nipah viral pathogenesis along with the transmission have been much well understood due to extensive research. This understanding is going to be more advanced in the decade to come. It is important to note in this regard that the practical utility of this understanding will be reflected in the entry of Nipah viral vaccines into clinical trials in humans, and modification of risk factors in order to prevent infection. Further, such understanding will be great aid in developing techniques along with therapeutics for treatment of infected subjects for reduction of morbidity as well as mortality. Prevention of such zoonotic disease in agricultural and healthcare workers should be a priority. Scientists have presented from platform like Global Outbreak Alert and Response Network (GOARN) especially after the outbreaks in Bangladesh and India and marked the necessity of development of network communicating between veterinary and medical services concerning this disease. By involving the multiple sectors and with multidisciplinary approach, precise and concrete preventive strategies can be planned and implemented.

The ‘One Health’ approach is also the utmost importance. There is requirement of coordination between institutes as well as at the international level among virologists from both medical and veterinary fields as well as ecologists for understanding to the fullest the period and mechanism involved in excretion of the virus by the bats. At the same time, the common people should be educated about food hygiene as well as hygiene at personal level. Inspection of all the imported livestock at the time of arrival and also before travel at the point of origin is essential. Proper isolation, quarantine and disinfection protocol including infrastructure facilities and trained personnel with protective clothing should be in place to respond quickly upon identification of any new case. There should be maintenance of proper hygiene at maximum level for slaughtering such livestock. For preventing future NiV outbreaks, a continuous surveillance in the area of human health, animal health, and reservoir hosts should be carried out to determine the prevalence and to predict risk of virus transmission in human and swine populations. Successful accelerated development of preventive vaccines and therapeutic antibodies or antivirals are need of the hour to control the spread and treat the infected patients during an outbreak. Collaborative efforts such as CEPI and biotech companies will accelerate the vaccine or therapeutic development for NiV.
